# Ethnobotanical study on medicinal plants used by Bulang people in Yunnan, China

**DOI:** 10.1186/s13002-023-00609-0

**Published:** 2023-09-07

**Authors:** Hao Zhou, Jiaqi Zhang, Brian S. Kirbis, Zi Mula, Wei Zhang, Yinzhi Kuang, Qing Huang, Lun Yin

**Affiliations:** 1 School of Geography and Ecotourism, Southwest Forestry University, Kunming, 650224 Yunnan China; 2 Center for Integrative Conservation, Xishuangbanna Tropical Botanical Garden, Chinese Academy of Sciences, Menglun, 666303 Yunnan China; 3 University of Chinese Academy of Sciences, Beijing, 100049 China; 4 Ministry of Education Key Laboratory for Ecology of Tropical Islands, Key Laboratory of Tropical Animal and Plant Ecology of Hainan Province, College of Life Sciences, Hainan Normal University, Haikou, 571158 Hainan China; 5 Jing Hong, China; 6 Xishuangbanna Ancient Tea Plant Conservation and Development Association, Jing Hong, 666100 Yunnan China; 7 Southwest Ecological Civilization Research Center, National Forestry and Grassland Administration, Kunming, 650224 Yunnan China

**Keywords:** Bulang people, Ethnobotany, Medicinal plants, Bulang traditional medicine, Traditional dai medicine (TDM)

## Abstract

**Background:**

Despite the popularity of modern medicine, medicinal plants remain a cornerstone of treatment for numerous diseases, particularly among ethnic groups and tribal communities around the globe. Ethnomedicine offers advantages such as ease of use, convenience, and economic benefits. Medicinal plant knowledge within Bulang ethnic community of southwest China is a valuable complement to Chinese ethnomedicine systems. Accumulated medical knowledge is due to the extensive length of occupation by Bulang People, considered the earliest inhabitants of Xishuangbanna; this has resulted in the development of various traditional treatment methods with local characteristics and unique curative effects. Therefore, there is exceeding value in exploring the medical knowledge of Bulang.

**Methods:**

A total of 175 local informants participated in the interviews and distribution of questionnaires in 10 Bulang villages in Menghai County, Xishuangbanna Prefecture, Yunnan Province, China. We documented the community of Bulang's use of medicinal herbs, and we used both the informant consensus factor (ICF) and use value (UV) methodologies to analyze the data. Furthermore, we conducted a comparative study to explore the potential of Bulang traditional medicine by comparing it to traditional Dai medicine.

**Results:**

The study recorded 60 medicinal plant species belonging to 41 families and 59 genera, including 22 species of herb, 22 species of shrub, nine species of trees, and seven species of liana. Araceae, Compositae, Lamiaceae and Leguminosae were found to have the highest number of species. The affordability and cultural heritage of Bulang medicine make it advantageous, Investigated Informants report that increased usage of Western medicine (88%), less availability of herbal medicine (95.43%), and the reduction in medicinal plant resources (80.57%) pose significant threats to Bulang medicine. All Bulang medicinal plants are naturally grown, with only 22 per cent being cultivated. *Camellia sinensis* (0.94) and *Zingiber officinale* (0.89) showed the highest UV values, while the function of *Phyllanthus emblica* L. and *Houttuynia cordata* Thunb. were also noted. The ICF revealed digestive system related diseases were the most commonly treated, with conditions of the motor system using the highest number of plant species. Finally, a comparison with traditional Dai medicine determined that 22 plants (36.67%) of the 60 surveyed had higher medicinal value in Bulang medicine.

**Conclusion:**

Bulang communities primarily source medicinal plants from the wild. Should environmental damage lead to the extinction of these medicinal plants, it could result in a shift toward modern Western medicine as a preferred medical treatment. Bulang ethnomedicine is a vital supplement to China's traditional medicine, particularly aspects of ethnic medicine relevant to daily life. Future research should emphasize inter-ethnic medical studies to reveal the untapped potential of medicinal plants.

## Background

International traditional medicine comprises various forms, such as Indian, Arabic, and Chinese; connecting different genres is essential to transmitting medicinal civilization and maintaining social relationships. Ayurveda, the predominant school of traditional Indian medicine, boasts a lengthy history in the Ganges Valley and has notably influenced South Asian traditional medicine. Traditional medicine in South Asia represents a traditional medicine system with Indian medicine serving as its core. During the Arabian era between the seventh and fourteenth centuries AD considered a romantic period in the history of Western medicine, the culture of therapy underwent significant development, playing a pivotal role in fostering later medicinal advancements. Traditional Chinese Medicine (TCM) maintains an inseparable connection with Arabic and Indian medicine, with these three primary therapeutic systems playing an integral role in transmitting human medicinal civilization and sustaining social relations [1, 2]. And within TCM, ethnic traditional medicine is an inseparable component. China is home to 55 ethnic minorities, each with unique traditional medicine that employs thousands of herbal remedies. Data indicates 12,087 kinds of traditional medicine resources in China, including 11,146 plants, 1,581 animals, 80 minerals, and more than 8,000 ethnic medicines, the Mongol, Tibetan, Uighur, and Dai ethnic medicine systems utilize at least 5,000 varieties of medicine. Ethnic medicine offers distinct advantages and holds great potential for treating cancer, bone setting, pain relief, rheumatism, psychiatric disorders, and the development of insect repellents and insecticides. Following Chinese scholars' classification of traditional medicine, there are three distinct categories. The first category comprises traditional ethnomedicinal knowledge systems with well-established written records, including medical codices, pharmacopoeias, and professional educational institutions that train physicians. Examples of such knowledge systems include Chinese, Mongolian, Tibetan, Uyghur, and Dai traditional medicines. The second category comprises traditional ethnomedicinal knowledge that has yet to form systematic pharmacopoeias and is mainly transmitted orally without formal professional institutions or organizations that provide training to physicians, including Yi, Miao, Hani, and Bulang medicine. The third category involves primitive or shamanic medicinal knowledge. Pharmacology in this category relies primarily on oral transmission and often incorporates psychological suggestion and supernatural sensing in herb-based disease treatments. Several ethnic groups in the Americas, including the Inuit, Oroqen, Ewenki, and Jinuo People, preserve this knowledge [3]. Bulang medicinal knowledge belongs to the second category, and the lack of literature on the restorative practices of the Bulang, both from domestic and foreign researchers, has been noted [4]. Historical literature indicates that the Bulang region was a breeding ground for various acute infectious diseases, known as the "land of miasma," where falciparum malaria was prevalent. Consequently, local herbalists would collect medicinal plants and provide treatment at home for nominal payment while also engaging in farming activities throughout the week. These folk doctors are called "Talaqi" by Bulang people [5, 6]. According to the data [7], the Bulang ethnic group in China has a reported total population of 136,782. Menghai is the largest settlement of the Bulang people in China.

In contrast with Traditional Dai Medicine (TDM), TDM is an ancient ethnic medicine system in China that has more than 2500 years of experience and has been collecting and organizing for years [8, 9]. Its approach has incorporated and integrated the practices of Indian medicine, Hinduism, Buddhism, and local wisdom to form a distinct medical theory. The Chinese government recognized TDM as one of the four primary ethnic medicines in China in 1984, alongside Tibetan, Mongolian, and Uyghur medicine. The extensive knowledge of conventional medicine among ethnic groups residing in the same region is considered an essential resource in ethnomedicine. In this study, we compared the medicinal plants utilized by Bulang ethnic group with those used by the Dai ethnic group. Previous research has compared TDM with TCM and Tai Medicine, but the comparison between the medicinal plants of Bulang and Dai has not been explored before [10–13], Bulang people have continuously summarized and absorbed the traditional medical knowledge of other ethnic minorities in the long-term relationship with other ethnic minorities, especially the Dai ethnic group, to improve and enrich the traditional medical experience of their own. However, Bulang people mainly live in mountainous areas while Dai people mostly live in flat land, and due to the geographical environment, the uses of the same plant of the two ethnic groups may be diverse.

Some scholars have made statistics on the development status and drug resources of Bulang Traditional medicine, but there is little relevant literature. Li et al. applied and analyzed the medicinal plant resources of six major ethnic minorities living in Xishuangbanna and recorded 49 kinds of Bulang medicinal plants[14], and 95 medicinal plants were cross used by other ethnic groups, with this high rate of coincidence, they believed that there was no significant difference in the types of diseases treated between ethnic groups, and most medicinal plants did not significantly differ in the types of diseases treated. Yang et al. believe that there is little research on the medical collation of Bulang people and a lack of written records. Although modern medical treatment has replaced ethnic medicine in the cities, it is still a crucial way of treating Bulang people in remote mountainous regions [15]. Yi et al. found a lack of Bulang medicine talents, low income, uncertainty about access to medicinal plants, and assimilation of Bulang medicine by TDM [16]. Zhang et al. researched traditional beliefs and oral health practices among Bulang people. They found they take herbal medicines internally and apply herbs and tobacco to relieve symptoms, such as pain, but no records of medicinal plants[17].

In summary, despite a few investigations into Bulang medicinal knowledge, the detailed records of Bulang medicinal plants and the comparative research with TDM still need to be included. The paper aims to record the therapeutic differences between the Bulang medicinal plants and compare the medicinal properties of the Dai and Bulang.

## Methods

### Study area

**Fig. 1:**
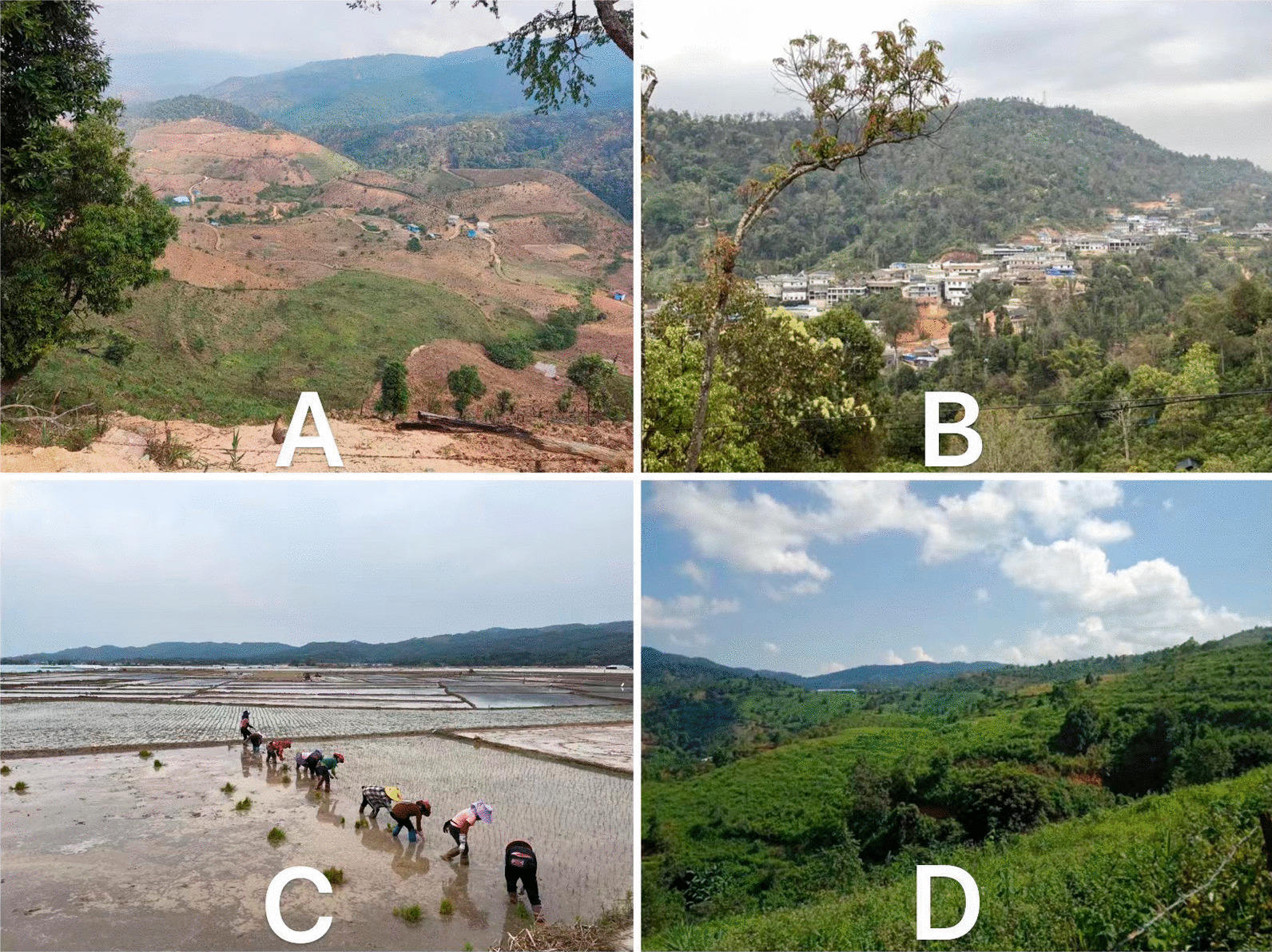
**A** Uncultivated wasteland in a Bulang village on high mountain; **B** A Bulang house complex in a deep valley; **C** A flat farmland at the foot of the Bulang settlement mountain; **D** Pu’er tea small tree in Bulang village

**Fig. 2:**
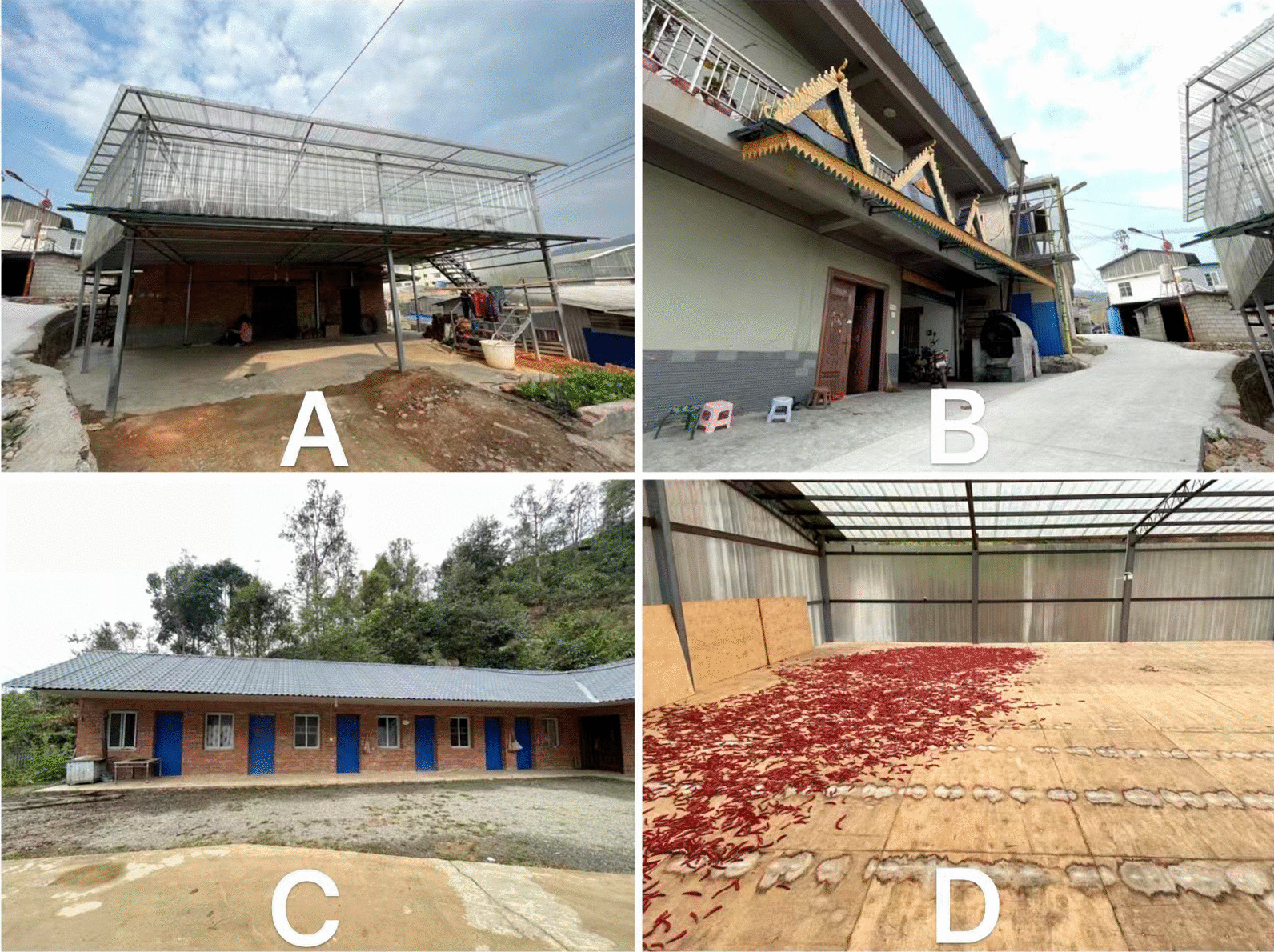
**A** Panoramic view of a double-story with diaphanous glass roof Bulang house; **B** The street view in a Bulang village; **C** A Bulang bungalow; **D** The top floor of a Bulang house is often used to dry crops such as tea or chili peppers

Bulang people primarily inhabit complex terrain characterized by high mountains and deep valleys, situated on both sides of the middle and lower reaches of the Lancang and Nujiang rivers, mainly south of 25°N latitude, ranging between 1500 and 2300 m above sea level during the post-fermentation process. Bulang people are primarily engaged in mountain agriculture, in sharp contrast to Dai people living in the flatland area (Fig. 1). Due to the influence of warm and humid air currents from the Indian Ocean and southwest monsoons, the climate varies considerably with elevation; the minimum temperature in winter is around 3–4 °C and the maximum temperature in summer reaches 30 °C, with an average annual temperature ranging between 19 and 22 °C. The region experiences a rainy season with high humidity and abundant rainfall from May to October. In contrast, the dry season is characterized by less rain and more fog from November to April. The natural environment provides suitable habitats for diverse flora and fauna. The traditional houses of Bulang people used to be divided into two-story wooden buildings, with the animals kept downstairs and firewood and agricultural tools piled up; people lived upstairs, and a fire pit was set up in the center of the house for cooking, heating, and lighting. However, with the development of China in recent decades, Bulang’s houses have been converted into modern cement houses, which are almost indistinguishable from ordinary Chinese rural dwellings in appearance and function apart from the translucent glass roof, which is installed for basking crops. The first floor of the two-story house is for living and cooking, and the second floor is for drying tea or chili peppers, peppercorns, herbs, etc. The Bulang bungalow is generally used only for living (Fig. 2). Pu’er tea, cultivated throughout the Bulang region, is particularly significant. Pu’er tea is a unique microbial fermented tea produced from the sun-dried leaves of large-leaf tea species *(Camellia sinensis (Linn.)* var. assamica (Masters) Kitamura) in the Yunnan province of China, has become increasingly popular in Southeast Asia may be due to its multiple health benefits. The unique sensory characteristics of Pu’er tea arise from the multitudinous chemical changes and transformations of the chemical constituents of the sun-dried green tea leaves that occur during the post-fermentation process[18]. Apart from tea plantation, Bulang people engage in subsistence cultivation of rice, corn, wheat, beans, buckwheat, sorghum and millet, followed by crops such as peanuts, sesame, sunflower, rape, pepper, cotton, ginger, tobacco and a variety of vegetables and edible mushrooms [19].

### Ethnobotanical data collection

**Fig. 3:**
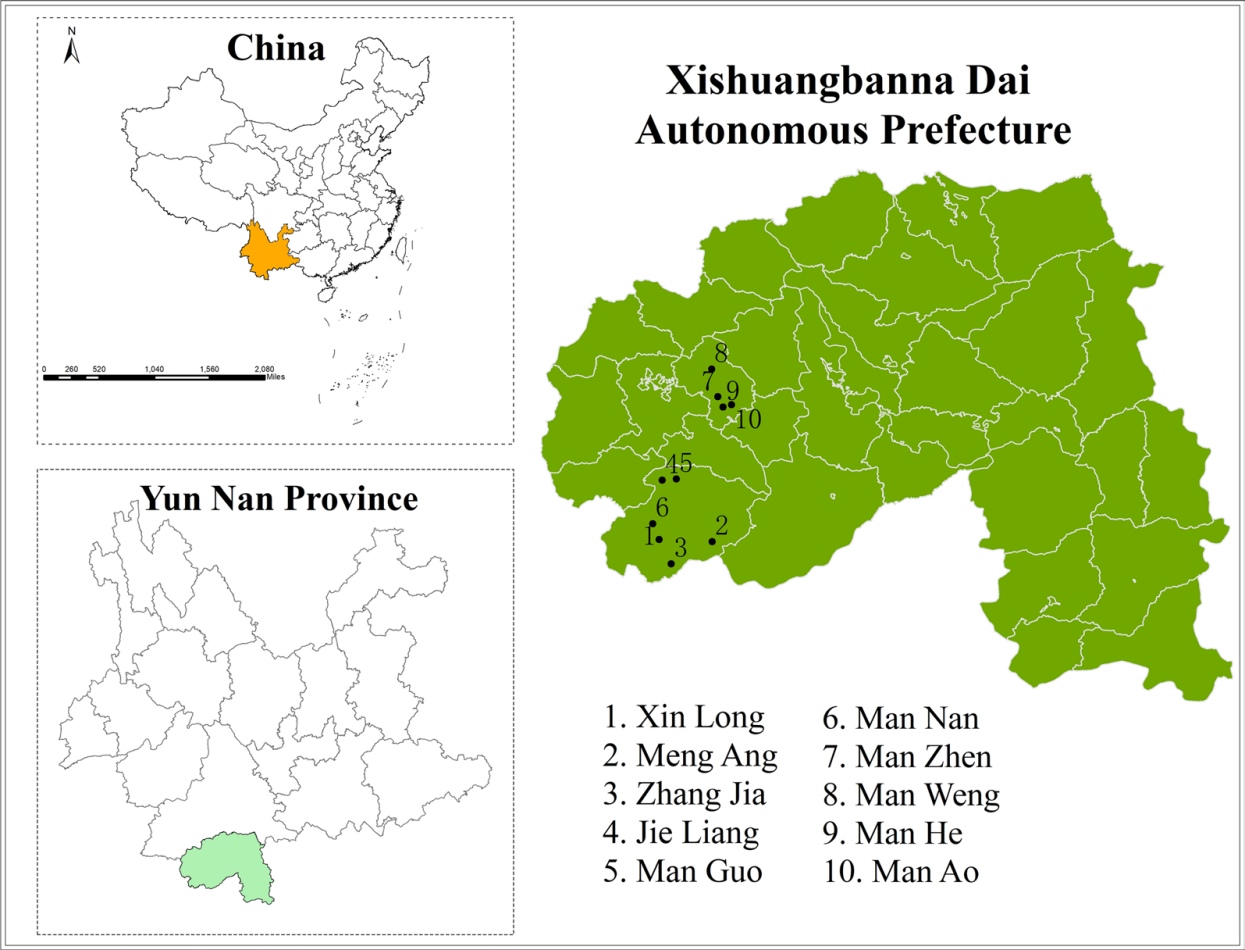
Map of study areas

**Fig. 4:**
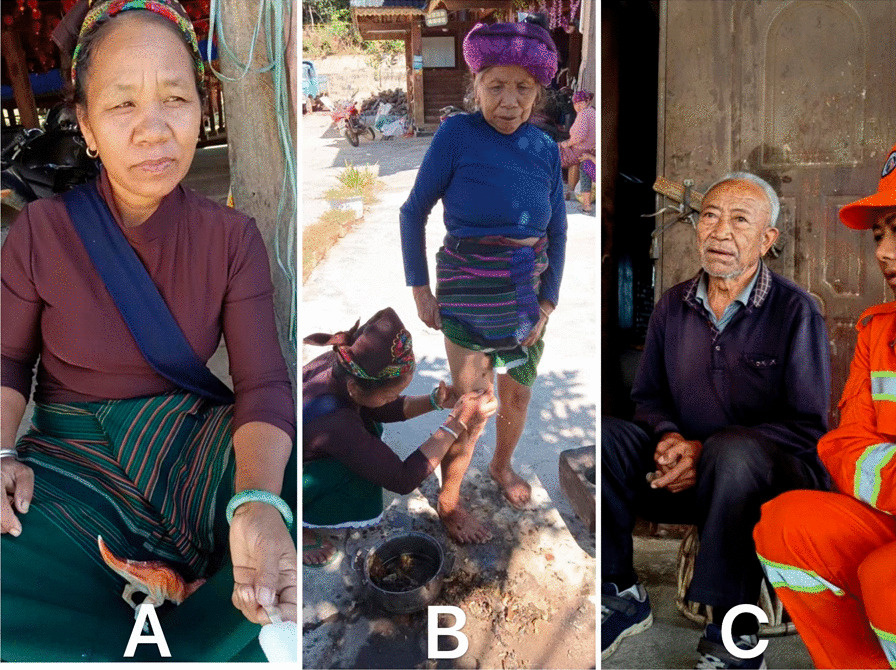
**A** A female Bulang folk doctor; **B** The Bulang doctor is applying herbs to an elderly's leg to relieve her leg pain; **C** A male Bulang folk doctor

We collected ethnobotanical data from November 2020 to February 2023 in 10 villages of Menghai County, Xishuangbanna Dai Autonomous Prefecture in Yunnan, China, including Xin Long, Meng Ang, Zhang Jia, Jie Liang, Man Guo, Man Nan, Man Zhen, Man Weng, Man He, Man Ao (Fig. 3). A total of 175 informants, consisting of 109 males and 66 females, was interviewed. Among the 175 informants, 43 key informants were Bulang folk doctors proficient in traditional medicine and were selected using the snowball method (Fig. 4). In comparison, the other 132 informants were users or information providers of Bulang medicine and without specialized knowledge. The study adhered to international ethical guidelines, and prior informed consent was obtained from each participant prior to interviews. To protect the intellectual property rights of the respondents, the study did not involve any discussion of confidential remedies. Verbal consent was obtained from each individual before their interview.

**Fig. 5:**
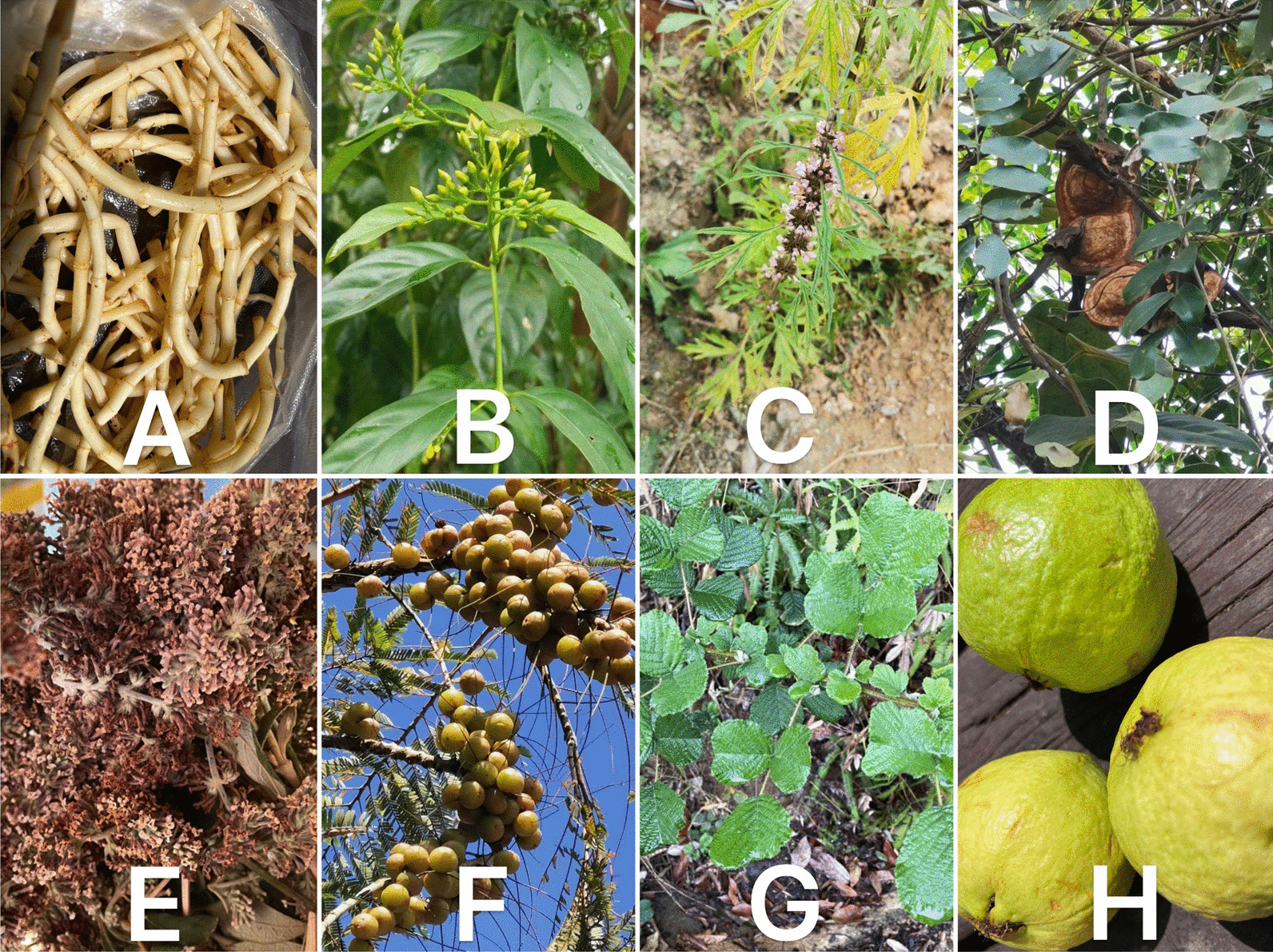
Fresh medicinal plants. **A** *Houttuynia cordata* Thunb., **B** *Gelsemium elegans* (Gardn. et Champ.) Benth., **C** *Leonurus japonicus* Houtt*.*, **D** *Entada phaseoloides* (L.) Merr., **E** *Buddleja officinalis* Maxim. **F** *Phyllanthus emblica L.* **G** *Rubus ellipticus* var. obcordatus (Franch.) Focke **H** *Psidium guajava* L

**Fig. 6:**
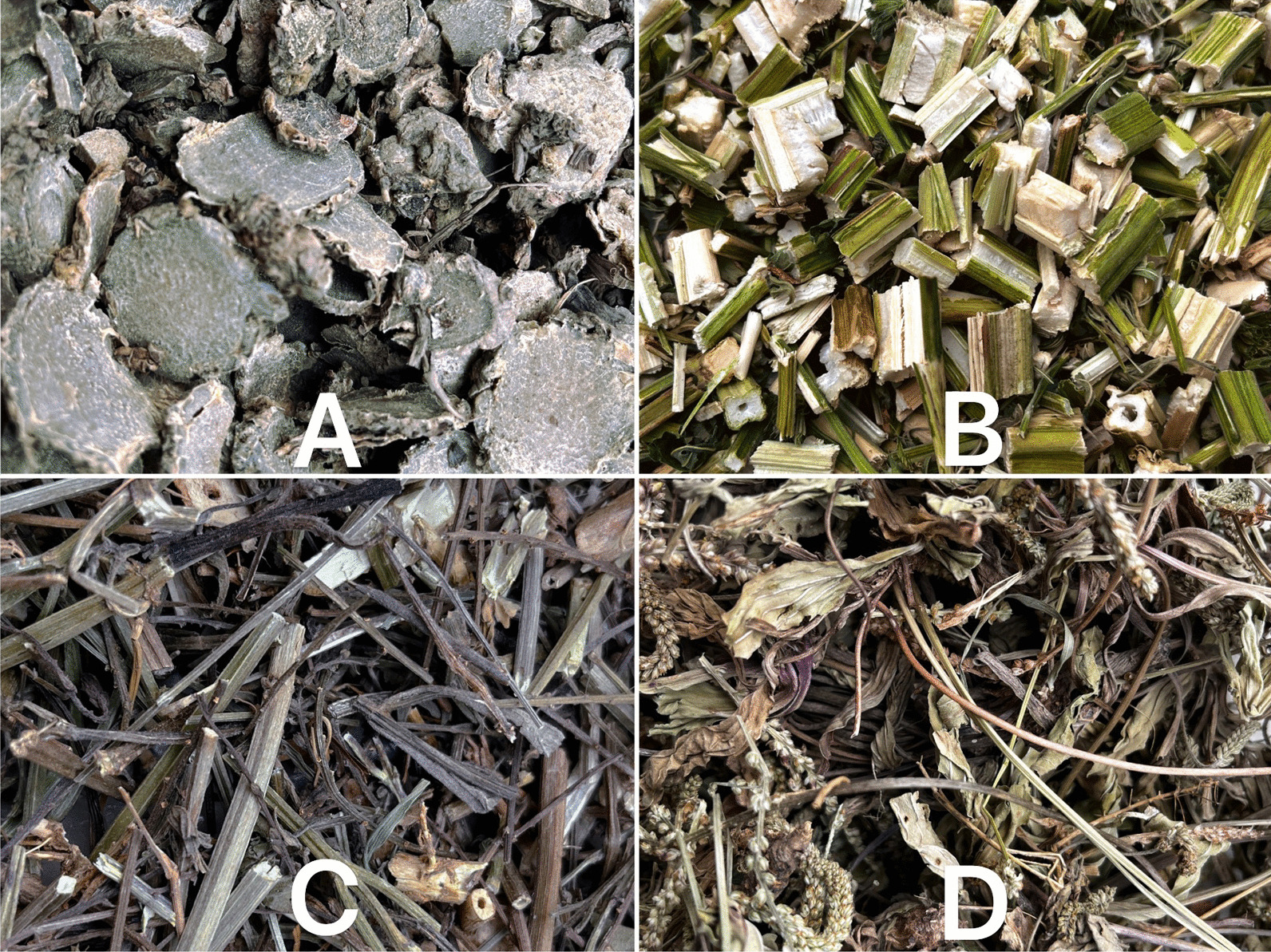
Dried plants **A** *Curcuma phaeocaulis* Valeton, **B** *Leonurus japonicus* Houtt., **C** *Verbena officinalis* L., **D** *Sambucus adnata* Wall. ex DC

**Fig. 7:**
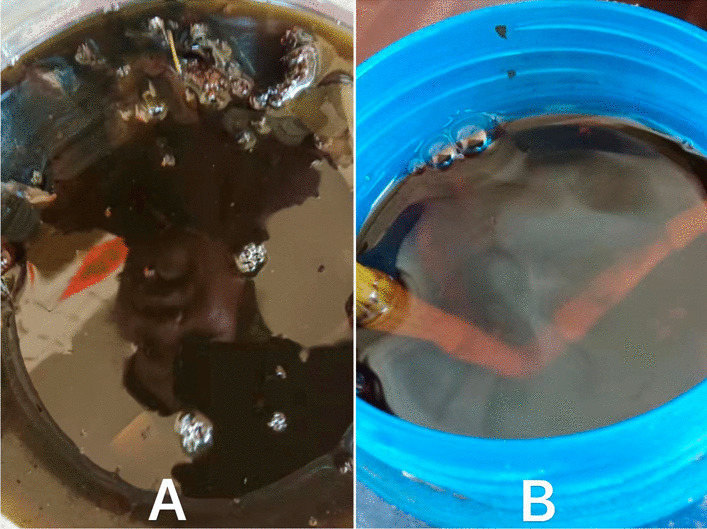
**A** & **B** Secret Bulang herbal remedies for rheumatism, detumescence and stasis

We employed unstructured and semi-structured interviews to gather information on the medicinal plants used by farmers and homebound elderly individuals with rudimentary Chinese language abilities. Before conducting the interviews, potential interviewees were identified through preliminary questioning. Translators were hired to facilitate communication and ensure accuracy. During the interview, we asked respondents two questions: Why do you choose a Bulang folk doctor when sick? What are the shortcomings or threats of Bulang traditional medicine? Demographic information, such as age and gender, was recorded for each participant, along with details on the local and scientific names of the plants used for medication, treatments administered, preparation methods, and parts utilized. Figures 5 and 6 are some medicinal plants, and Fig. 7 is secret Bulang herbal remedies in the study area. Botanist Dr. Zhang Jiaqi from the Center for Integrative Conservation, Xishuangbanna Tropical Botanical Garden, Chinese Academy of Sciences, confirmed the identification of each plant to complete the list of medicinal plants. The plant specimens were cited from the Xishuangbanna Tropical Botanical Garden Herbarium (HITBC).

### Data analysis

In 1986, American botanist Robert T. Trotter introduced the Informant Consensus Factors (ICF) to examine variability in data obtained from ethnobotanical field surveys. The ICF is defined as the degree of variation in the number of medicinal plant species utilized by doctors in treating a particular type of disease. $ICF=\frac{Nur-Nt}{Nur-1}$, where Nur is the number of references used for each category and Nt is the number of species used, the value of ICF ranging from 0 to 1, with higher values indicating greater consensus among informants [20, 21].

Use Value (UV) is a valuable tool for assessing the significance of local species. It reflects the frequency of use for a given species among the informants, with Ui representing the number of uses reported by each informant and Ut being the total number of informants: $UV=\frac{Ui}{Ut}$. As initially introduced by Prance in 1987, UV is calculated as the sum of a species' primary and secondary use values within a particular culture [22], this approach has been widely adopted in ethnobotanical studies to identify the most important species within a given population. The UV metric ranges from zero to a positive value, with higher UV values indicating greater importance of a species and lower UV values indicating less importance [23, 24].

## Results

### Informant demographics and questionnaire survey

The study comprised 175 informants (Table 1), which displays statistics on age, gender, and occupation, along with a rationale for the benefits and risks associated with Bulang medicine. Participant ages ranged from 19 to 84 years old, with the majority (70%) being over 30. The male-to-female ratio was 2:1, and over 86% of the participants were local farmers whose primary livelihood was growing cash crops such as rice and tea. Regarding Reasons for selecting Bulang traditional medicine when ill, respondents provided four primary responses: (1) cost-effectiveness in comparison with Western medical practices; (2) significance of Bulang medicine as a cultural legacy; (3) presence of three to five herbalists in each village making local treatment more accessible than traveling to a hospital; and (4) the effectiveness of traditional Bulang medicine, surpassing that of Western medicine.

**Table 1:** Demographic features of informants

Demographic features		Number	Proportion(%)
*Sex*			
	Male	107	61.14
	Female	68	38.86
*Age*			
	10–20	9	5.14
	21–30	42	24.00
	31–40	65	37.14
	41–50	22	12.57
	51–60	17	9.71
	61 and above	20	11.43
*Vocation*			
	Farmer	151	86.29
	Civil servant	18	10.29
	Student	6	3.43
*Reason for using traditional Bulang medicine*			
	Cost-effective	147	84.00
	Cultural heritage	145	82.86
	Accessible	81	46.29
	Positive efficacy	87	49.71
*Threats to traditional Bulang medicine*			
	Fewer traditional doctors	167	95.43
	Western medicine cures more diseases	154	88.00
	Medicinal plant resource reduction	141	80.57
	Unpalatable taste	134	76.57

When queried the current drawbacks or challenges of Bulang medicine, participants raised the following concerns: (1) the declining number of Bulang herbalists (95.43%); (2) the limited range of diseases that Bulang medicine can treat compared to Western medicine (88%); (3) the declining availability of medicinal plants due to environmental degradation (80.57%); (4) the unpalatable taste and difficulty in swallowing Bulang herbal medicine in contrast to Western medicine tablets (76.57%).

### Medicinal plants recorded

**Fig. 8:**
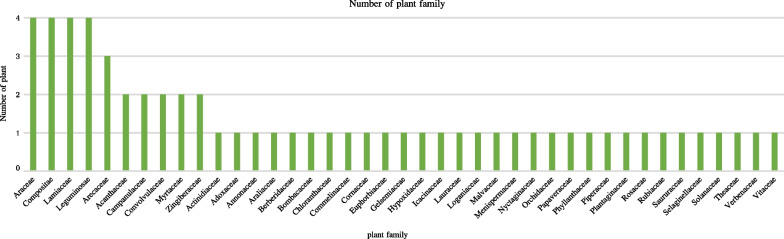
Family of investigated medicinal plants

**Fig. 9:**
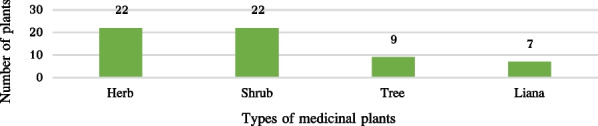
Types of investigated medicinal plants

**Fig. 10:**
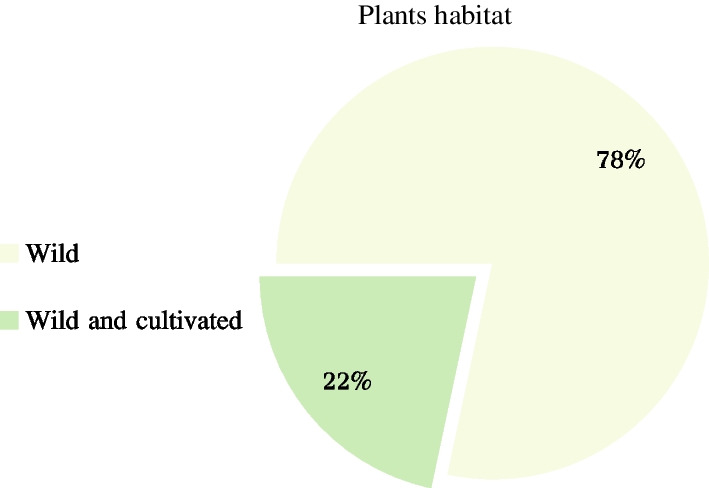
Habitats of investigated plants

A total of 60 species belonging to 41 families and 59 genera of medicinal plants are identified, for which scientific name, Chinese name, Bulang name, family, habit, use value, habitat, parts used, medicinal use, and disease treatment for each species are all provided (Tables 2 and 3, Fig. 8). Notably, Araceae, Compositae, Lamiaceae and Leguminosae plants were the most commonly encountered species among the study population. The identified species were further categorized into four types, with 22 herbs, 22 shrubs, nine trees, and seven lianas (Fig. 9). Nearly all of the medicinal plants utilized by Bulang were wild-sourced, mainly in natural habitats such as mountains, streams, and roadsides, with 22% of the plants being cultivated (Fig. 10).

**Table 2:** List of medical plants used by Bulang People

No	Scientific name	Chinese name	Bulang name (phonetic)	Family	Genus	Habit	Habitat	UR	UV	NDT	Cited sources (HITBC)
1	*Acorus calamus* L	Changpu菖蒲	Hengkawu	Araceae	Acorus	Herb	Wild	53	0.3	3	HITBC0023758
2	*Alocasia cucullata* (Lour.) G.Don	Jianweiyu尖尾芋	Layin	Araceae	Alocasia	Herb	Wild	70	0.4	1	HITBC0035032
3	*Areca catechu* L	Binglang槟榔	Duai	Arecaceae	Areca	Tree	Wild and cultivated	62	0.36	1	HITBC0057782
4	*Argyreia osyrensis* (Roth) Choisy	Huimaobaiheteng灰毛白鹤藤	Pengsuke	Convolvulaceae	Argyreia	Shrub	Wild	18	0.11	4	HITBC0023164
5	*Argyreia wallichii* Choisy	Dayeyinbeiteng 大叶银背藤	Gewake	Convolvulaceae	Argyreia	Liana	Wild	21	0.12	4	HITBC0031152
6	*Artemisia annua* L	Huanghuahao黄花蒿	Niangmuhin	Compositae	Artemisia	Herb	Wild	51	0.29	1	HITBC0023019
7	*Bombax ceiba* L	Mumian木棉	gennemniu	Bombacaceae	Bombax	Tree	Wild	20	0.11	1	HITBC0032598
8	*Buddleja officinalis* Maxim	Mimenghua密蒙花	Sagong	Loganiaceae	Buddleja	Shrub	Wild and cultivated	120	0.69	4	HITBC0068684
9	*Callerya cinerea* (Bentham) Schot	Huimaojixueteng灰毛鸡血藤	Che	Leguminosae	Callerya	Shrub	Wild	94	0.54	4	HITBC0026784
10	*Camellia sinensis* var. assamica (J. W. Masters) Kitam	Pu'er cha 普洱茶	La	Theaceae	Camellia	Tree	Wild and cultivated	165	0.94	6	HITBC0078335
11	*Chloranthus spicatus* (Thunb.) Makino	Jinsulan金粟兰	Teng	Chloranthaceae	Chloranthus	Shrub	Wild	64	0.36	3	HITBC0078567
12	*Clerodendrum bungei* Steud	Choumudan臭牡丹	Yayinhe	Lamiaceae	Clerodendrum	Shrub	Wild	59	0.34	2	HITBC0058215
13	*Cryptocoryne crispatula* var. yunnanensis (H. Li) H. Li & N. Jacobsen	Baxianguohai八仙过海	Gawa	Araceae	Cryptocoryne	Herb	Wild	38	0.22	5	HITBC0069233
36	*Curculigo capitulata (Lour.) O. Kuntze*	Dayexianmao大叶仙茅	Songsenga	Hypoxidaceae	Molineria	Herb	Wild	37	0.21	2	HITBC0078571
14	*Curcuma phaeocaulis* Valeton	Ezhu莪术	Kuomin	Zingiberaceae	Curcuma	Herb	Wild and cultivated	36	0.21	3	HITBC0076473
15	*Cyanotis arachnoidea* C. B. Clarke	Zhusimaolanercao蛛丝毛蓝耳草	Luopueng	Commelinaceae	Cyanotis	Herb	Wild	33	0.19	1	HITBC0078686
16	*Dactylicapnos scandens* (D. Don) Hutch	Zijinlong紫金龙	Niasabang	Papaveraceae	Dactylicapnos	Liana	Wild	40	0.23	1	HITBC0047398
17	*Datura metel* L	Yangjinhua洋金花	Pengpusuoke	Solanaceae	Datura	Shrub	Wild and cultivated	71	0.4	3	HITBC0023142
18	*Duhaldea cappa* (Buch.-Ham. ex D.Don) Pruski & Anderb	Yangerju羊耳菊	Giaoen	Compositae	Inula	Shrub	Wild	21	0.12	1	HITBC0058053
19	*Eclipta prostrata* (L.) L	Lichang鳢肠	Geyouen	Compositae	Eclipta	Herb	Wild and cultivated	89	0.51	1	HITBC0061932
20	*Elaeis guineensis* Jacq	Youzong油棕	Dewa	Arecaceae	Elaeis	Tree	Wild and cultivated	18	0.1	1	HITBC0035156
21	*Eleutherococcus trifoliatus* (Linnaeus) S.Y.Hu	Baile白簕	Dangjieli	Araliaceae	Eleutherococcus	Shrub	Wild	65	0.37	2	HITBC0079320
22	*Entada phaseoloides* (L.) Merr	Keteng榼藤	Songbue	Leguminosae	Entada	Liana	Wild	23	0.13	1	HITBC0059666
23	*Fissistigma polyanthum Merr*	Heifengteng黑风藤	Zao	Annonaceae	Fissistigma	Shrub	Wild	35	0.2	5	HITBC0040475
24	*Flemingia macrophylla* (Willd.) Merr	Dayeqianjinba大叶千斤拔	Niasabang	Leguminosae	Flemingia	Shrub	Wild	20	0.11	1	HITBC0033081
25	*Gelsemium elegans* (Gardn. et Champ.) Benth	Gouwen钩吻	Hebugenye	Gelsemiaceae	Gelsemium	Liana	Wild	36	0.2	2	HITBC0058566
26	*Helwingia japonica* (Thunb.) Dietr	Qingjiaye青荚叶	Lake	Cornaceae	Helwingia	Shrub	Wild	87	0.5	3	HITBC0068037
27	*Homalomena pendula* (Blume) Bakh. f	Daqiannianjian大千年健	Yayinhen	Araceae	Homalomena	Herb	Wild	63	0.36	3	HITBC0023785
28	*Houttuynia cordata* Thunb	Jicai蕺菜	Pakadong	Saururaceae	Houttuynia	Herb	Wild and cultivated	169	0.97	4	HITBC0047123
29	*Iteadaphne caudata* (Nees) H. W. Li	Xiangmianye香面叶	Chuche	Lauraceae	Iteadaphne	Shrub	Wild	18	0.1	4	HITBC0015570
30	*Justicia adhatoda* L	Yazuihua鸭嘴花	Yasangduo	Acanthaceae	Justicia	Shrub	Wild	34	0.19	3	HITBC0065488
31	*Leonurus japonicus* Houtt	Yimucao益母草	Yamuhin	Lamiaceae	Leonurus	Herb	Wild and cultivated	117	0.67	1	HITBC0076156
32	*Lobelia clavata* E. Wimm	Mimaoshangengcai密毛山梗菜	bengfa	Campanulaceae	Lobelia	Shrub	Wild	52	0.3	3	HITBC0023106
33	*Mahonia bealei* (Fortune) Pynaert	Kuoyeshidagonglao阔叶十大功劳	wa,gewate	Berberidaceae	Mahonia	Shrub	Wild	34	0.2	1	HITBC0020293
34	*Mappianthus iodoides* Hand.-Mazz	Dingxinteng定心藤	Kuoya	Icacinaceae	Mappianthus	Liana	Wild	99	0.56	1	HITBC0060007
35	*Mirabilis jalapa* L	Zimoli紫茉莉	Wailing	Nyctaginaceae	Mirabilis	Herb	Wild and cultivated	25	0.14	1	HITBC0070744
38	*Phyllanthus emblica* L	Yuganzi余甘子	Beme	Euphorbiaceae	Phyllanthus	Tree	Wild	170	0.97	7	HITBC0021234
39	*Phyllanthus reticulatus* Poir	Xiaoguoyexiazhu小果叶下珠	Longle	Phyllanthaceae	Phyllanthus	Shrub	Wild	57	0.33	1	HITBC0069180
40	*Piper boehmeriifolium* (Miq.) Wall. ex C.DC	Zhuyeju苎叶蒟	Delu	Piperaceae	Piper	Shrub	Wild	21	0.12	6	HITBC0075144
41	*Plantago asiatica* L	Cheqian车前	Yayinnen	Plantaginaceae	Plantago	Herb	Wild	40	0.23	6	HITBC0060231
42	*Pogostemon glaber* Benth	Ciruicao刺蕊草	Saigong	Lamiaceae	Pogostemon	Herb	Wild	36	0.21	1	HITBC0060306
43	*Premna szemaoensis* C.Pei	Simaodoufucai思茅豆腐柴	Pengsuo	Lamiaceae	Premna	Tree	Wild	120	0.68	6	HITBC0079397
44	*Psidium guajava* L	Fanshiliu番石榴	Magui	Myrtaceae	Psidium	Shrub	Wild and cultivated	44	0.25	4	HITBC0078248
45	*Rubus ellipticus* var. obcordatus (Franch.) Focke	Zaiyangbiao栽秧藨	Gacai	Rosaceae	Rubus	Shrub	Wild	47	0.27	4	HITBC0070806
46	*Sambucus adnata* Wall. ex DC	Xuemancao血满草	Niasabang	Adoxaceae	Sambucus	Herb	Wild	37	0.21	3	HITBC0062395
47	*Saurauia napaulensis DC*	Niboershuidongge尼泊尔水东哥	Langgai	Actinidiaceae	Saurauia	Tree	Wild	6	0.03	3	HITBC0058528
48	*Schizomussaenda henryi* (Hutch.) X. F. Deng et D. X. Zhang	Lieguojinhua 裂果金花	Luopuei	Rubiaceae	Schizomussaenda	Shrub	Wild	93	0.53	2	HITBC0026054
49	*Selaginella pulvinata* (Hook. et Grev.) Maxim	Dianzhuangjuanbai垫状卷柏	Gewa	Selaginellaceae	Selaginella	Herb	Wild	17	0.1	1	HITBC0026683
50	*Stephania epigaea* H.S. Lo	Diburong地不容	Gemeng	Menispermaceae	Stephania	Liana	Wild	96	0.55	1	HITBC0078464
51	*Strobilanthes cusia* (Nees) Kuntze	Banlan板蓝	Heigenyin	Acanthaceae	Strobilanthes	Herb	Wild	84	0.48	5	HITBC0077607
52	*Syzygium globiflorum* (Craib) Chantaran. & J.Parn	Duanyaoputao短药蒲桃	Gemeng	Myrtaceae	Syzygium	Shrub	Wild	98	0.56	1	HITBC0020744
53	*Tadehagi triquetrum* (L.) Ohashi	Hulucha葫芦茶	Gewape	Leguminosae	Tadehagi	Shrub	Wild	41	0.24	1	HITBC0068568
54	*Tetrastigma hemsleyanum* Diels et Gilg	Sanyeyapateng三叶崖爬藤	Songlong	Vitaceae	Tetrastigma	Liana	Wild	16	0.09	6	HITBC0058579
55	*Thunia alba* (Lindl.) Rchb. F	Sunlan笋兰	Gawape	Orchidaceae	Thunia	Herb	Wild	53	0.3	4	HITBC0078770
56	*Trachycarpus fortunei* (Hook.) H. Wendl	Zonglv棕榈	Mangbengku	Arecaceae	Trachycarpus	Tree	Wild and cultivated	7	0.04	1	HITBC0077678
57	*Urena lobata* L	Ditaohua地桃花	Gemeng	Malvaceae	Urena	Herb	Wild	19	0.11	4	HITBC0029213
58	*Verbena officinalis* L	Mabiancao马鞭草	Hongsenga	Verbenaceae	Verbena	Herb	Wild	80	0.46	2	HITBC0062712
37	*Vernonia parishii* Hook. f	Dianmianbanjiuju滇缅斑鸠菊	Bengfa	Compositae	Monosis	Tree	Wild	19	0.11	3	HITBC0060661
59	*Wahlenbergia marginata* (Thunb.) A. DC	Lanhuashen蓝花参	Yayinhia	Campanulaceae	Wahlenbergia	Herb	Wild	51	0.29	1	HITBC0068901
60	*Zingiber officinale* Roscoe	Jiang姜	Gagin	Zingiberaceae	Zingiber	Herb	Wild and cultivated	155	0.89	9	HITBC0031289

NDT = Number of diseases treated, UV = Use value, UR = Use report

**Table 3:** Methods of use for reported medicinal plants

No	Scientific name	Parts used	Preparation	Application	Ailment category description	Therapeutic uses (therapeutic use report)
1	*Acorus calamus*	Root, stem, leaf	Decocted in water, chew	Oral	D,R,M	Abdominal pain (29), cold (11), detumescence (36)
2	*Alocasia cucullata*	whole plant	Decocted in water with brown sugar	Oral	C	Heart disease (70)
3	*Areca catechu*	Flower	Decocted in water	Oral	E	Diabetes (62)
4	*Argyreia osyrensis* var. cinerea Hand.-Mazz	Root	Decocted in water	Oral, external washing	G	Irregular menstruation (12), mastitis (8), uterine prolapse (9)
5	*Argyreia pierreana*	Root	Decocted in water	Oral, external washing	G	Irregular menstruation (13), mastitis (17), uterine prolapse (16), prolapse of anus (5)
6	*Artemisia annua*	Root, leaf	Decocted in brown sugar water	Oral	D	Dysentery (51)
7	*Bombax ceiba*	Leaf, skin of fruit	Pounded	Eternal application	M	Fracture (20)
8	*Buddleja officinalis*	Flower, leaf	Decocted in water	Oral	R,O	Cough (49), asthma (23), eye disease (26), pharyngitis (105)
9	*Callerya cinerea*	Root, stem	Decocted in water	Oral	C,G,M	Stimulating blood circulation (69), detumescence (83), irregular menstruation (37)
10	*Camellia sinensis*	Leaf	Infused in water, cook	Oral	D,R,C	Abdominal distension (56), cold (89), cough (94), enteritis (39), heat clearing and detoxification (121), pharyngitis (67)
11	*Chloranthus spicatus*	whole plant	Decocted in water, pounded	Oral, external application	M,I	Detumescence (15), rheumatism (22), fracture (23)
12	*Clerodendrum bungei*	Root	Decocted in water	Oral	I,A	Rheumatism (16), analgesia (48)
13	*Cryptocoryne crispatula* var. yunnanensis	whole plant	Decocted in water	Oral	M,I,D	Detumescence (17), rheumatism (19), enteritis (29), stomachache (12)
14	*Curculigo capitulata*	Root	Decocted in water	Oral	A,I	Analgesia (24), rheumatism (28)
15	*Curcuma phaeocaulis*	whole plant	Decocted in water	Oral	D,I,M	Rheumatism (11), abdominal distension (30), detumescence (21)
16	*Cyanotis arachnoidea*	whole plant	Cook with pork	Oral	I	Rheumatism (33)
17	*Dactylicapnos scandens*	Root	Decocted in water	Oral	C	Anemia (40)
18	*Datura metel*	whole plant	Pounded	Eternal application	A,M	Analgesia (45), fracture (31), detumescence (25)
19	*Duhaldea cappa*	whole plant	Decocted in water	Oral	Gs	Cystitis (21)
20	*Eclipta prostrata*	whole plant	Decocted in water	Oral	D	Abdominal pain (89)
21	*Elaeis guineensis*	Fruit	Decocted in water	Oral	E	Diabetes (18)
22	*Eleutherococcus trifoliatus*	whole plant	Decocted in water, cook	Oral or external application	R	Parotitis (65)
23	*Entada phaseoloides*	Seed	Pounded	External application	O	Sore (23)
24	*Fissistigma polyanthum*	Stem	Infused in water, Decocted in water	Oral, external application	D,M	Invigorating spleen (9), stimulating blood circulation (27), detumescence (12), fracture (21)
25	*Flemingia macrophylla*	Root	Decocted in water	Oral	G	Irregular menstruation (20)
26	*Gelsemium elegans*	Root	Infused in water	External washing	O,M	Sore, (23) detumescence (25)
27	*Helwingia japonica*	whole plant	Decocted in water, pounded	Oral, external application	M,C	Fracture (33), stimulating blood circulation (43), detumescence (32)
28	*Homalomena pendula*	Root, stem	Infused in water, Decocted in water	Oral	R,I	Fever (40), tuberculosis (24), bronchitis (29)
29	*Houttuynia cordata*	whole plant	Infused in water	External washing	O,R,I	Cold (87), cough (142), fever (39), sore (141)
30	*Iteadaphne caudata*	Root, leaf, bark	Decocted in water, powdered, pounded	Oral, external application	A,M	Analgesia (15), hemostasis (10), detumescence (9), fracture (8)
31	*Justicia adhatoda*	Bark, Branch	Pounded	Eternal application	A,M	Fracture (12), analgesia (19), cough (10)
32	*Leonurus japonicus*	whole plant	Decocted in water, infused in water	Oral, external washing	G	Irregular menstruation (117)
33	*Lobelia clavata*	Root	Infused in water, Decocted in water and liquor	Oral	R,M,I	Parotitis (22), detumescence (28), rheumatism (12)
34	*Mahonia bealei*	Root	Decocted in water	Oral	C	Heat clearing and detoxification (34)
35	*Mappianthus iodoides*	whole plant	Decocted in water	Oral	C	Heart disease (34)
36	*Mirabilis jalapa*	Root	Decocted in water	Oral	Gs	Prostatitis (25)
37	*Phyllanthus emblica*	Fruit, stem	Decocted in water	Oral	D,R,C	Pharyngitis (143), abdominal distension (56), abdominal pain (92), cough (120), heat clearing and detoxification (87)
38	*Phyllanthus reticulatus*	Fruit	Decocted in water	Oral	E	Diabetes (57)
39	*Piper boehmeriifolium*	whole plant	Decocted in water	Oral	R,D,G,M	Cold (8), detumescence (12), rheumatism (3), stomachache(9), dysmenorrhea (5)
40	*Plantago asiatica*	Whole plant	Decocted in water	Oral	Gs,R,C	Urinary retention (11), leucorrhea (37), hematuria (21), cough (7), pharyngitis (12), heat clearing and detoxification (32)
41	*Pogostemon glaber*	whole plant	Decocted in water	Oral	D	Enteritis(36)
42	*Premna szemaoensis*	Root, bark	Decocted in water, powdered	Oral, external application	C,A,M,I	Stimulating blood circulation (97), analgesia (69), hemostasis (78), fracture (97), detumescence (97), rheumatism (26)
43	*Psidium guajava*	Leaf, fruit	Decocted in brown sugar water, pounded	Oral, external application	D,M,A	Enteritis(29), dysentery (11), detumescence (21), hemostasis (23)
44	*Rubus ellipticus Smith* var. obcordatus	Root	Decocted in water	Oral	D,I	Diarrhea (41), enteritis (34), dysentery (15), rheumatism (22)
45	*Sambucus adnata*	Whole plant	Decocted in water	Oral	Gs,I,M	Nephritis (12), rheumatism (9), fracture (21)
46	*Saurauia napaulensis*	Skin of fruit	Decocted in water	Oral, external application	M,A	Detumescence (6), fracture (6), hemostasis (3)
47	*Schizomussaenda henryi*	Bark	Decocted in water	Oral	R,C	Pharyngitis (42), heat clearing and detoxification (64)
48	*Selaginella pulvinata*	whole plant	Infused in water	Oral	G	Dystocia (17)
49	*Stephania epigaea*	Leaf	Powdered	Oral	D	Stomachache (96)
50	*Strobilanthes cusia*	Root, leaf	Decocted in water	Oral	R,D,C,I	Parotitis (10), amygdalitis (59), stomatitis (21), dysentery (32), heat clearing and detoxification (52)
51	*Syzygium globiflorum*	Bark	Decocted in water	Oral	D	Food poisoning (98)
52	*Tadehagi triquetrum*	Root	Decocted in water	Oral	E	Diabetes (41)
53	*Tetrastigma hemsleyanum*	Root	Infused in liquor, powdered	Oral, external application	I,A,C	Analgesia (10), hemostasis (12), stimulating blood circulation (15), detumescence (14), fracture (8)
54	*Thunia alba*	whole plant	Decocted in water, pounded	Oral, external application	I,M,R	Detumescence (21), cough (39), rheumatism (23), fracture (41)
55	*Trachycarpus fortunei*	Root	Decocted in water	Oral	E	Diabetes (7)
56	*Urena lobata*	Root	Decocted in water	Oral	R,A,I	Cold (12), hemostasis (17), rheumatism (9), heat clearing and detoxification (14)
57	*Verbena officinalis*	Whole plant	Decocted in water	Oral	R,I	Cold (80), fever (80)
58	*Vernonia parishii*	Root	Decocted in water	Oral	G,I,D	Postpartum care (9), rheumatism (18), hepatitis (12)
59	*Wahlenbergia marginata*	Whole plant	Decocted in water	Oral	D	Stomachache (51)
60	*Zingiber officinale*	Root and stem	Pounded, cook	Oral, external application	R,D,M,C	Cold (145), cough (96), asthma (60), abdominal distension (78), detumescence (87), fracture (102), abdominal pain (42), pharyngitis (89), heat clearing and detoxification (116)

Ailment category description: A = Analgesia, C = Circulatory system, D = Digestive system, E = Endocrine diseases, G = Gynecology, Gs = Genitourinary system, I = Immune system, M = Motor system, O = Other uses, R = Respiratory

### Use value

We utilized the use report (UR) to calculate the use value (UV) of the reported medicinal plants, providing a means to assess their relative importance in the study area and shed light on the preferred medicinal plants used by Bulang people (Table 2). The UV values of *Phyllanthus emblica* L. and *Houttuynia cordata* Thunb. were both found to be 0.97, indicating their significance in local practice. Additionally, *Camellia sinensis* var. assamica (J. W. Masters) Kitamura (0.94) and *Zingiber officinale* Roscoe (0.89) were also among the plants with high UV values. In contrast, *Tetrastigma hemsleyanum* Diels et Gilg (0.09), *Trachycarpus fortunei* (Hook.) H. Wendl. (0.04), and *Saurauia napaulensis* DC. (0.03) had the lowest recognition for their medicinal properties.

### Preparation and application

**Fig. 11:**
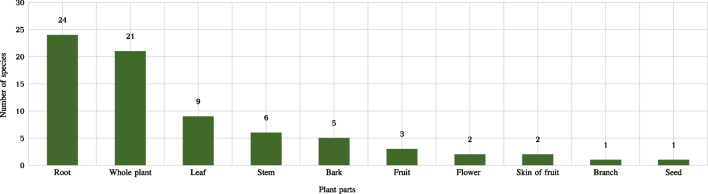
Parts utilized

Table 3 presents medicinal plant parts that are utilized in traditional Bulang medicine preparation. Bulang people employ whole plant, branch, seed, flower, skin, bark, stem, leaf, and root to formulate medicinal concoctions (Fig. 11). The root is the most frequently used, with 24 plant species (40%). Meanwhile, 21 plant species (35%) employ the whole plant for medicinal purposes. The least utilized plant parts are the seed and branch (1.6%).

Six primary methods are used in the preparation of traditional medicinal plants by Bulang people. The Decoction is The most common method, accounting for 80% of all preparations. This method involves medicinal components in fresh, sugar or alcohol. Following decoction, the most prevalent methods include pounding (16.67%) and infusing (15%), typically involving steeping in water or alcohol. The remaining methods include powdering (6.67%), cooking (6.67%), and chewing (1.67%). Oral application was the most commonly utilized (54 species, 73%), followed by external application (15 species, 20%) and medicinal washing (5 species, 7%).

### Informant consensus factor

We systematically categorized distinct symptoms based on human body systems disease systems, identified ten unique groupings of symptoms and subsequently determined the number of illnesses that fell within each classification (Table 4). The type of diseases in this paper is based on actual treatment results and human body systems. Based on the survey and records conducted in the study area, 41 diseases were treated with medicinal plants. Subsequent statistical analysis revealed that ailments associated with the digestive system were the most commonly treated afflictions. Nine distinct digestive diseases, including enteritis, abdominal pain, and abdominal distension, could be addressed using investigated plants, with 26 plant species identified as viable remedies. The motor system was another frequently treated domain, with 31 plants employed to treat conditions such as fracture and detumescence. All ICF values were reported to be more than 0.9; the highest is other use (sore and eye disease) (0.986), followed by the respiratory system (0.985), circulatory system (0.984), endocrine diseases (0.978), motor system (0.969), analgesia (0.968), immune system (0.964), digestive system (0.963), gynecology (0.962) and genitourinary system (0.962).

**Table 4:** Informant consensus factor by categories of investigated areas

Disease category	Ailments	Number of ailments	Nur	Nt	ICF
Analgesia	Analgesia(7), hemostasis(6)	2	373	13	0.968
Circulatory system	Anemia(1), stimulating blood circulation(5), heat clearing and detoxification(8), heart disease(2)	4	915	16	0.984
Digestive system	Enteritis(5), abdominal pain(4), abdominal distension(4), hepatitis(1), stomatitis(1), dysentery(4), stomachache(4), food poisoning(1), invigorating spleen(1)	9	651	25	0.963
Endocrine diseases	Diabetes(5)	1	185	5	0.978
Genitourinary system	Cystitis(1), hematuria(1), prostatitis(1), nephritis(1)	4	79	4	0.962
Gynecology	Postpartum care(1), leucorrhea(1), dystocia(1), mastitis(2), prolapse of anus(1), irregular menstruation(5), uterine prolapse(2)	7	317	13	0.962
Immune system	Rheumatism(13), amygdalitis(1), fever(3)	3	450	17	0.964
Motor system	Fracture(13), detumescence(18)	2	978	31	0.969
Other uses	Sore(3), eye disease(1)	2	213	4	0.986
Respiratory	Tuberculosis(1), cold(7), cough(8), pharyngitis(6), parotitis(2), asthma(1), bronchitis(1)	7	1658	26	0.985

## Discussion

### Threats to traditional medicinal knowledge and medicinal plants

As per the results of the informants' interview, the majority of individuals familiar with Bulang traditional medicine fell between the ages of 30 and 60 (83.43%), with this age group demonstrating a higher level of definitive knowledge regarding medicinal plants than other age cohorts [25–28]. Interviews on the question of “What are the shortcomings or threats of Bulang traditional medicine?” indicated that 95% of Bulang perceived the declining number of folk doctors as one of the major factors impeding the progress of Bulang medicine. The main reason for this perception may be that Bulang folk doctors rarely practice medicine as a professional occupation, and their diagnostic fees are usually inexpensive. Revenue generated from medical practice is not a primary source of income for Bulang folk practitioners. The inheritance of traditional Bulang medicine manifests in diverse ways, with transmission occurring through familial channels, self-guided learning, experiential practice, accumulation of knowledge, and collection of medicinal preparations. Due to the lack of a written tradition, the origin and information related to the treatment procedures are not documented. Some Bulang practitioners have acquired medical knowledge from their ancestors through Dai language manuscripts, including family medical books and scriptures that cannot be shared with outsiders and are limited to male family members. Dai language belongs to the Zhuang-Dai branch of the Zhuang-Dong group of the Chinese-Tibetan Phylum, or family of languages. Dai has a writing system, which is written in an alphabetic instead of a character script. As ethnic medicine culture continues to evolve, Bulang practitioners seek to enhance their proficiency by studying Dai and Hani traditional medicinal knowledge [29, 30]. Dai and Hani villagers rely on forests for food and medicine, and most villagers and traditional healers retain some traditional knowledge of medicinal plants, which is more comprehensively documented and compiled. Bulang doctor’s consultation fees vary depending on the patient's origin. For individuals from the same village, a modest price of only 6–7 RMB is charged per visit, and sometimes, these services are provided free of charge, with ritual candles offered as an act of goodwill under their Theravada Buddhist beliefs. Conversely, those from other regions outside the province, such as Beijing and Shanghai, are charged nearly 100 RMB per visit. Diagnostic assessments by Bulang practitioners generally involve patient self-reporting, pulse-taking, and facial observation, similar to the diagnostic methods employed in TDM and TCM, which include observing, questioning, listening, smelling, and palpating.

In addition, the survey results revealed that a significant proportion (88%) of participants perceived modern medicine as more efficacious in treating diseases than traditional medicine. As China's education rate has increased in recent decades, individuals may increasingly value modern medicine's scientific underpinnings and express concerns regarding the potential adverse side effects of herbal medicine. Moreover, 80.57% of interviewees expressed the availability of medicinal plants is declining. While not all Bulang people may be practicing herbalists, they are generally knowledgeable about the flora of their surroundings since they need to differentiate between edible and poisonous plants. Unfortunately, the number of medicinal plants used by Bulang people is limited, and there is no active cultivation of these plants. The rapid changes in the environment and habitat destruction make it increasingly difficult for herbalists to find medicinal plants in the wild, which could lead to the discontinuation of their use or a reduction in their efficacy, ultimately causing patients to turn to Western medicine. This lack of sustained access to medicinal plants represents a significant challenge to the continuity of Bulang medicine.

Socioeconomic changes could result in losing or reducing medicinal plants and related indigenous knowledge [31]. Researchers have proved that a decline in medicinal plants may hinder the development of traditional medicine [32–34]. Xishuangbanna boasts exceptional biodiversity, positioning it among the world's most affluent regions. Nonetheless, human activities such as the under-forest economy and rubber plantation have resulted in an alarming loss of biodiversity in the area. While 41.7% of forests in the uplands (i.e., above 900 masl.) are located in the altitudinal zone of 900–1200 masl., the rapid expansion of rubber plantations into higher elevations, steeper terrain and nature reserves poses a severe threat to biodiversity and environmental services, resulting in a loss of agrobiodiversity while not producing the expected economic returns [35–38]. Rubber and tea collections have become the dominant agricultural activities from March to November and February to October, respectively. Tea production in Bulang Mountain Township surpassed 2,888 tons by the end of 2021, generating a total output value of 1.9 billion yuan [39–41]. The availability of medicinal plants in the Bulang community has declined due to wild collection and the reduction or loss of knowledge and cultivation practices. Inheritance of ethnomedicine and socioeconomic changes have contributed to this decline, also evident in the shrinking Bulang gardens. The tea economy and urbanization have led to the rebuilding of houses that occupy more space, leaving less room for medicinal plants. As a result, growing medicinal plants for profit was never a priority, and they are rarely sold as modern pharmacies have become prevalent in towns. This phenomenon is not unique to the Bulang community but rather a common issue associated with the loss of traditional knowledge and the decline in biodiversity due to development [42].

More than 80% of the survey participants emphasized the cultural significance of Bulang medicine, viewing it as a crucial aspect of Bulang ethnic identity. The development and evolution of traditional medicinal knowledge among ethnic minorities have been significantly shaped by the interplay of cultural, historical, environmental, and belief systems. These communities highly value traditional medicine knowledge, considering it a significant cultural heritage with deep cultural roots [43]. In ethnic minority groups, traditional medicine is more than just a treatment method; it symbolizes cultural identity, a source of community pride, and an integral aspect of the social fabric. These findings underscore the importance of preserving and promoting traditional medicinal knowledge to protect cultural heritage and promote sustainable development. The preservation of traditional medicinal knowledge is an essential aspect of safeguarding and propagating minority cultures. Various groups, including the government, scholars, communities, and knowledge bearers, are working together to protect the endangered traditional medicine culture. These collaborative efforts focus on documenting and safeguarding traditional knowledge, providing training and education to knowledge bearers and younger generations, and creating strategies for the future development of this valuable knowledge [44–46].

### Use value and ICF

Upon analyzing the dataset for Use Value, the two botanical specimens with the greatest reported usage were *Phyllanthus emblica* and *Houttuynia cordata*, ascertaining their significant ethnobotanical value (0.97). *Phyllanthus emblica* belongs to the *Phyllanthus* genus of the *Phyllanthaceae* family and is extensively distributed across subtropical and tropical regions in countries such as China, India, Indonesia, and Malaysia. Its fruits are known to have high concentrations of vitamin C and superoxide dismutase, exhibiting hepatoprotective, antibacterial, anticancer, and anti-inflammatory properties [10–13]. *Phyllanthus emblica* has been documented in traditional Bulang and Dai medicine for treating various ailments. Bulang medicine employs *Phyllanthus emblica* to treat liver and gallbladder diseases, pharyngitis, abdominal distension, abdominal pain, cough, scurvy, heat clearing and detoxification, liver and gallbladder disorders, pharyngitis, abdominal distension, abdominal pain, cough, scurvy, stopping itching, sores, fever, cough etc.

*Houttuynia cordata* is a widely distributed and highly esteemed edible plant in southwestern China, highly regarded and consumed by Dai, Bulang, Lahu, Hani, Yao, and Dong ethnic groups [47, 48]. Using plants as both natural medicines and food sources presents a promising avenue for exploring new dietary supplements with potentially lower human safety risks and improved health outcomes [49]. Therefore, integrating *Houttuynia cordata* into modern food systems may significantly improve human health and well-being. *Saurauia napaulensis*, with the lowest Use value, is primarily distributed in southeastern and southwestern Yunnan, southwestern and northwestern Guang Xi, Gui Zhou, as well as in India, Nepal, Myanmar, Laos, Thailand, Vietnam, and Malaysia. It thrives in mountainous areas, sparse forests, and thickets situated at an altitude range of approximately 500-1500 m. Despite its extensive distribution, there needs to be more research on this plant, domestically or internationally, with only a few studies examining its chemical composition [50, 51]. It is used for detumescence, fracture, and hemostasis in both Dai and Bulang medicine.

With 165 individuals reporting its medicinal value, *Camellia sinensis* var. Assamica scores a high Use value of 0.94. Herbal beverages are consumed for recreational or therapeutic purposes [52–55]. Tea is the second most consumed beverage after water, with the global average per capita consumption of boiled tea being 120 ml per day [56]. Pu'er tea, the local product, is a distinct, fermented variety of tea made from the sun-dried leaves of *Camellia sinensis var. assamica*, endemic to Yunnan, China. The characteristic brown hue of the tea leaves is a result of microbial fermentation by *Aspergillus niger* during processing, in conjunction with the action of leaf oxidase [57]. Research indicates that this fermented tea exhibits a plethora of biological activities, including but not limited to antioxidant, antimutagenic, antibacterial, laxative, neuroprotective, anti-hypercholesterolemic, anti-hyperglycemic, anti-obesity, anti-diabetic, anti-osteoporotic, and anti-Alzheimer's properties, as well as inhibitory effects against fungi, cancer, and inflammation [58–61]. Notably, research also highlights the presence of certain undesired chemicals, such as heavy metals and mycotoxins, with the growing, processing and storage conditions of tea plantations being closely associated with such health concerns [62].

Bulang people use Pu'er tea as both food and medicine. Ubiquitous are Paste Rice Tea and Ming Zi Tea. Paste Rice Tea is prepared by baking glutinous rice in an earthen teapot until it turns yellow and then adding tea leaves, boiled water, sliced ginger, and brown sugar. It is believed to have therapeutic properties that help alleviate colds, coughs, sore throats, heat, dry lungs, and other ailments. In addition, Ming Zi tea is made similarly to paste rice tea but with pine resin, a sticky substance secreted by pine trees, a combination of natural oils from pine and wood fibers. The different parts of the pine and cypress trees have varying oil content, with the roots containing the highest levels and the higher branches having lower levels. This tea is believed to help alleviate gastrointestinal discomfort, constipation, and other related conditions. Another unique tea consumption method is Sour Tea, which involves fermented tea leaves. Rather than being brewed with boiling water, Sour Tea is chewed directly, allowing its flavor and aroma to fill the mouth while promoting digestion, quenching thirst, and generating fluids.

### Comparison of Dai and Bulang’s applications of investigated plants

Before comparing the two ethnomedicines, understanding the difference in living altitude created a boundary between the two groups is essential. Dai, who inhabit the plains, historically referred to Bulang people living in the mountains as Man or Ka, meaning 'mountain-dweller' and 'slave', respectively. Xishuangbanna has traditionally been more economically advantageous for the Dai than the Bulang. This advantage was based on the pattern of Dai occupying the more agriculturally accessible lowlands. Nevertheless, Bulang people practiced subsistence cultivation in mountainous areas, trading tea and other substances in local periodic markets. However, inter-ethnic relations have undergone significant changes with the establishment of modern market systems and the focus on market economics. In particular, the combined efforts of foreign capital and the local resources of Pu'er tea have led to a change in the relationship between the Bulang and Dai [63, 64].

Current ethnic medicine narratives emphasize TDM's formal acknowledgement as one of China's four traditional medicines, but Bulang medicine has yet to be thoroughly investigated and structured. When Theravada Buddhism first appeared in Xishuangbanna in 1437, the Dai written language was primarily intended to preserve and transmit Buddhist teachings. Bulang people, who lacked written language, occasionally adopted the Dai script through their conversion to Buddhism. Consequently, research on Bulang medicine is still in its nascent stage due to the absence of written records; with scarce ancient literature dedicated to the subject matter and few references to Bulang medicine in other historical texts, oral transmission remains the primary mode of preserving and transmitting the existing traditional medicine knowledge among the Bulang ethnic group [65–67].

After analyzing the research data, we find 31 medicinal plants possess a greater therapeutic spectrum in TDM compared to Bulang, seven plants exhibit an equivalent therapeutic range in both ethnic groups (Table 5), while the remaining 22 plants listed in Table 6 display a higher degree of disease curability in Bulang medicine than in TDM. There are notable differences in the uses of specific plants between Dai and Bulang traditional medicines. *Psidium guajava*, for example, is commonly employed in TDM for heat clearing, detoxification, and skin conditions. In contrast, Bulang medicine primarily treats gastrointestinal ailments like enteritis, dysentery, and hemostasis. This highlights medicinal plants' unique approaches and applications in the two ethnic therapies. This plant finds applications for treating diarrhea, dysentery, diabetes, cardiovascular disease, cancer, parasitic infections, gastroenteritis, hypertension, diabetes, caries, pain relief and improvement in locomotor coordination. Previous research indicates that *Psidium guajava* is commonly used to produce essential oils with antibacterial, anti-inflammatory, mosquito-repellent, and wound-healing properties[68–70]. These findings highlight the potential of this plant as a multipurpose resource in ethnic medicine research.

**Table 5:** Comparison of Dai and Bulang applications of investigated plants

Chinese name & Scientific name	Ethnic group	Ethnic name	Parts used	Ailments
白簕 *Eleutherococcus trifoliatus*	B	Dang jie li	whole plant	Laryngitis, parotitis
	D	Gai dang	whole plant, root, leaf	Hypertension, cough, hyperlipidemia, cold, fever, emphysema
番石榴 *Psidium guajava*	B	Magui	Leaf, fruit	Enteritis, dysentery, detumescence, hemostasis
	D	Maguixiangla	Fruit, skin, leaf	Heat clearing and detoxification, dermatomycosis
大千年健 *Homalomena pendula*	B	Yayinhen	Root, stem	Fever, tuberculosis, bronchitis
	D	Pokou	Stem	Fever, tuberculosis, cold, rheumatism
栽秧藨 *Rubus ellipticus Smith* var. *obcordatus*	B	Gacai	Root	Diarrhea, enteritis, dysentery, rheumatism
	D	Mahulengying	Root, leaf	Detumescence, analgesia, amygdalitis, dysentery, sore, irregular menstruation
莪术 *Curcuma phaeocaulis*	B	Kuomin	whole plant	Rheumatism, abdominal distension, detumescence
	D	wanhainao	Root skin, stem skin	Nephritis, rheumatism
血满草 *Sambucus adnata*	B	Niasabang	whole plant	Nephritis, rheumatism, fracture
	D	Yashaban	Root, whole plant	Rheumatism, detumescence
车前 *Plantago asiatica*	B	Yayinnen	whole plant	Urinary retention, leucorrhea, hematuria, cough, pharyngitis, heat clearing and detoxification
	D	Pokou	Root, stem	Fever, tuberculosis, cold, rheumatism
黄花蒿 *Artemisia annua*	B	Niangmuhin	Root, leaf	Dysentery
	D	Yamaimen	Whole plant	Malaria
板蓝 *Strobilanthes cusia*	B	Heigenyin	Root, leaf	Parotitis, amygdalitis, stomatitis, dysentery, heat clearing and detoxification
	D	Menghuang	Whole plant, root	Heat clearing and detoxification, dizziness, analgesia
八仙过海 *Cryptocoryne crispatula* var*. yunnanensis*	B	Gawa	Whole plant	Detumescence, rheumatic arthritis, rheumatism, enteritis, stomachache
	D	Baxianguohai	Whole plant	Rheumatism, enterogastritis
苎叶蒟 *Piper boehmeriifolium*	B	Delu	Whole plant	Influenza, cold, detumescence, rheumatism, stomachache, dysmenorrhea
	D	Daidun	Whole plant, root	Detumescence, fracture, sore, cough, pneumonia
滇缅斑鸠菊 *Vernonia parishii*	B	Bengfa	Root	Postpartum care, rheumatism, hepatitis
	D	Elengluo	Whole plant, root, leaf	Detumescence, rheumatism, fracture, dermatomycosis
密毛山梗菜 *Lobelia clavata*	B	bengfa	Root	Parotitis, detumescence, rheumatism
	D	Biaobengfa	Root, leaf	Heat clearing and detoxification, sore, abdominal distension, rheumatism, lumbar muscle strain
思茅豆腐柴 *Premna szemaoensis*	B	Pengsuo	Root, bark	Stimulating blood circulation, analgesia, hemostasis, fracture, detumescence, rheumatism
	D	Yamaimen	Whole plant	Malaria, tuberculosis
黑风藤 *Fissistigma polyanthum*	B	Zao	Stem,root	Invigorating spleen, stimulating blood circulation, detumescence, fracture
	D	Guangmaodai	Root, stem	Rheumatism, cold, irregular menstruation, detumescence, fracture
尼泊尔水东哥 *Saurauia napaulensis*	B	Langgai	Skin of fruit	Detumescence, fracture, hemostasis
	D	Meiqimo	Skin	Detumescence, fracture, hemostasis
金粟兰 *Chloranthus spicatus*	B	Teng	Whole plant	Detumescence, rheumatism, fracture
	D	Pahuai	Whole plant, stem	Detumescence, fever, cold
青荚叶 *Helwingia japonica*	B	Lake	Whole plant	Fracture, stimulating blood circulation, detumescence
	D	Heilingniang	Seed, seed skin, stem, root, skin	Fever, heat clearing and detoxification
香面叶 *Iteadaphne caudata*	B	Chuche	Root, leaf, bark	Analgesia, hemostasis, detumescence, fracture
	D	Yasanying	Root, leaf, skin	Rheumatism, detumescence, analgesia
笋兰 *Thunia alba*	B	Gawape	Whole plant	Detumescence, cough, rheumatism, fracture
	D	Dangna	Root stem	Heat clearing and detoxification, urinary tract infection
洋金花 *Datura metel*	B	Pengpusuoke	Whole plant	Analgesia, fracture, detumescence
	D	Yahangyan	Whole plant, leaf, root	Cold, parotitis, urinary tract infection
木棉 *Bombax ceiba*	B	gennemniu	Leaf, skin of fruit	Fracture
	D	Biaobengfa	Root, leaf	Heat clearing and detoxification, parotitis, sore, abdominal distension, inappetence
菖蒲 *Acorus calamus*	B	Hengkawu	Root, stem, leaf	Abdominal pain, cold, detumescence
	D	Shabupu	Root stem	Hepatitis
垫状卷柏 *Selaginella pulvinata*	B	Gewa	Whole plant	Dystocia
	D	Molemao	Whole plant, root, fruit	Heat clearing and detoxification, detumescence
臭牡丹 *Clerodendrum bungei*	B	Yayinhe	Root	Rheumatism, analgesia
	D	Zhehanfang	Root, whole plant	Fever, cervicitis, detumescence
鸭嘴花 *Justicia adhatoda*	B	Yasangduo	Bark, Branch	Fracture, analgesia, cough
	D	Meishaomiao	Root skin, stem skin	Fracture, rheumatism
灰毛鸡血藤 *Callerya cinerea*	B	Che	Root, stem	Stimulating blood circulation, detumescence, irregular menstruation, amenorrhea
	D	Luoheng	Whole plant	Fracture, pneumonia
尖尾芋 *Alocasia cucullata*	B	Layin	Whole plant	Heart disease
	D	Yasanying	Root, leaf, skin	Analgesia, fracture, rheumatism
密蒙花 *Buddleja officinalis*	B	Sagong	Flower, leaf	Cough, asthma, eye disease, pharyngitis
	D	Mohaoleng	Bud, inflorescence	Hepatitis
马鞭草 *Verbena officinalis*	B	Hongsenga	Whole plant	Cold, fever
	D	Yahangyan	Whole plant, leaf, root	Cold, parotitis, urinary tract infection
蕺菜 *Houttuynia cordata*	B	Pakadong	Whole plant	Cold, cough, fever, sore
	D	Gebake	Root, leaf, flower, fruit, seed	Detumescence, heat clearing and detoxification
阔叶十大功劳 *Mahonia bealei*	B	Gewate	Root	Heat clearing and detoxification
	D	Lanhanduolan	Whole plant	Heat clearing and detoxification, diuresis, irregular menstruation, dysmenorrhea
余甘子*Phyllanthus emblica*	B	Beme	Fruit, stem	Liver and gallbladder diseases, pharyngitis, abdominal distension, abdominal pain, cough, scurvy, heat clearing and detoxification
	D	Maxiang	Leaf, root, fruit, skin	Pruritus, sore, fever, cough
鳢肠*Eclipta prostrata*	B	Geyouen	whole plant	Abdominal pain
	D	Mahulengying	Root, leaf	Detumescence, analgesia, dysentery, sore, irregular menstruation
大叶银被藤 *Argyreia wallichii*	B	Gewake	Root	Irregular menstruation, mastitis, uterine prolapse, prolapse of anus
	D	Yaxiaomang	Root, leaf	Mastitis, uterine prolapse, cough
三叶崖爬藤*Tetrastigma hemsleyanum*	B	Songlong	Root	Hemostasis, stimulating blood circulation, detumescence, fracture, and relieve pain
	D	Zhehanfang	Root	Detumescence
榼藤 *Entada phaseoloides*	B	Songbue	Seed	Sore
	D	Heilingniang	Seed, root, fruit skin	Fever, sore, amygdalitis
地桃花 *Urena lobata*	B	Gemeng	Root	Cold, hemostasis, rheumatism, heat clearing and detoxification
	D	Hanmannuosuo	Seed	Malaria, abdominal distension
灰毛白鹤藤 *Argyreia osyrensis* var*. cinerea*	B	Pengsuke	Root	Irregular menstruation, mastitis, uterine prolapse, rectocele
	D	Guodanggai	Root. stem, leaf	Heat clearing and detoxification, rheumatism
钩吻 *Gelsemium elegans*	B	Hebugenye	Root	Sore, detumescence
	D	Eluoleng	Root. stem, leaf	Heat clearing and detoxification, rheumatism, fracture
紫金龙 *Dactylicapnos scandens*	B	Niasabang	Root	Anemia
	D	Yalaihanfang	Root	Heat clearing and detoxification
槟榔 *Areca catechu*	B	Gema	Flower	Diabetes
	D	Gemabu	Root	Cough, rheumatism, heat clearing and detoxification
棕榈 *Trachycarpus fortunei*	B	Mangbengku	Root	Diabetes
	D	Geguo	Root	Hemostasis
油棕 *Elaeis guineensis*	B	Dewa	Fruit	Diabetes
	D	Yahanmansuoluo	Root. stem, leaf	Cold, rheumatism, heat clearing and detoxification, dysentery
小果叶下珠 *Phyllanthus reticulatus*	B	Longle	Fruit	Diabetes
	D	Dengheihan	Vine	Detumescence, urinary retention
葫芦茶 *Tadehagi triquetrum*	B	Gewape	Root	Diabetes
	D	Yahezhu	Root, whole plant	Heat clearing and detoxification, cold, fever
地不容 *Stephania epigaea*	B	Gemeng	Leaf	Stomachache
	D	Bomoying	Leaf, skin, stem	Rheumatism, analgesia, sore, parotitis
大叶仙茅 *Curculigo capitulata*	B	Songsenga	Root	Analgesia, rheumatism
	D	Danhuoma	Root. stem, leaf	Sore, rheumatism, heat clearing and detoxification
蛛丝毛蓝耳草 *Cyanotis arachnoidea*	B	Luopueng	whole plant	Rheumatism
	D	Yanghelang	Root. stem	Tuberculosis, cough, rheumatism
短药蒲桃 *Syzygium globiflorum*	B	Gemeng	Bark	Food poisoning
	D	Haoming	Stem	Rheumatism, irregular menstruation, sore
紫茉莉 *Mirabilis jalapa*	B	Wailing	Root	Prostatitis
	D	Meidian	Root	Rheumatism, irregular menstruation, detumescence
裂果金花 *Schizomussaenda henryi*	B	Luopuei	Bark	Pharyngitis, heat clearing and detoxification
	D	Dangna	Root, stem	Hepatitis, sore, urinary retention
定心藤 *Mappianthus iodoides*	B	Kuoya	Whole plant	Palpitation
	D	Huangjiu	Whole plant	Fever, abdominal pain, sore
刺蕊草 *Pogostemon glaber*	B	Saigong	Whole plant	Enteritis
	D	Guomainiu	Root, stem skin	Cough, postpartum care, constipation
蓝花参 *Wahlenbergia marginata*	B	Yayinhia	Whole plant	Stomachache
	D	Maiximo	Root, stem skin	Fracture, detumescence, urolithiasis
大叶千斤拔 *Flemingia macrophylla*	B	Niasabang	Root	Irregular menstruation
	D	Mohahao	Root, leaf	Detumescence, abdominal pain, rheumatism
益母草 *Leonurus japonicus*	B	Yamuhin	Whole plant	Irregular menstruation
	D	Nahan	Root	Abdominal pain, cold, fever
羊耳菊 *Duhaldea cappa*	B	Giaoen	Whole plant	Cystitis
	D	Mahangbang	Stem skin, fruit	Jaundice, dermatomycosis, cough
普洱茶 *Camellia sinensis* var*. assamica*	B	La	Leaf	Abdominal distension, cold, cough, enteritis, heat clearing and detoxification, pharyngitis
	D	Yashuaiyang	Whole plant	Stomachache, dysmenorrhea, rheumatism, detumescence
姜 *Zingiber officinale*	B	Yela	Leaf	Cold, cough, asthma, abdominal distension, detumescence, fracture, abdominal pain, pharyngitis, heat clearing and detoxification
	D	Xin	Stem, leaf	Detumescence, cold, urinary tract infection, cough, dysmenorrhea

^*^Ethnic groups B = Bulang People, D = Dai People

**Table 6:** List of 22 plants which cure more diseases in Bulang medicinal knowledge

Chinese name & Scientific name	
番石榴 *Psidium guajava*	鸭嘴花*Justicia adhatoda*
莪术*Curcuma phaeocaulis*	灰毛鸡血藤*Callerya cinerea*
血满草*Sambucus adnata*	密蒙花*Buddleja officinalis*
车前*Plantago asiatica*	蕺菜*Houttuynia cordata*
板蓝*Strobilanthes cusia*	余甘子*Phyllanthus emblica*
八仙过海*Cryptocoryne crispatula* var*. yunnanensis*	大叶银被藤*Argyreia wallichii*
思茅豆腐柴*Premna szemaoensis*	三叶崖爬藤*Tetrastigma hemsleyanum*
青荚叶*Helwingia japonica*	地桃花*Urena lobata*
香面叶*Iteadaphne caudata*	灰毛白鹤藤*Argyreia osyrensis* var*. cinerea*
笋兰*Thunia alba*	茶*Camellia sinensis*
菖蒲*Acorus calamus*	姜*Zingiber officinale*

In addition, there are several other plants worth discussing. *Entada phaseoloides* is a plant commonly used in traditional Bulang and Dai medicine to treat soreness, fever, and amygdalitis. Recent studies have revealed its use in Chinese Yao ethnic medicine to treat rheumatism, as a nutritional supplement, and to promote blood circulation [71]. Another notable observation is that *Callerya cinerea* and *Argyreia synesis var. cinerea*, both included in Bulang medicine, are purported to have therapeutic effects on gynecological ailments. However, this curative property needs to be mentioned in TDM or widely acknowledged in current research on these plants in China and abroad. *Eclipta prostrata* is recognized for its medicinal value in treating abdominal pain in Bulang medicine. However, in TDM, this plant is also used to treat detumescence, analgesia, dysentery, soreness, and irregular menstruation, as well as for liver protection, immunity regulation, and detoxification. These therapeutic effects have been verified through relevant studies [72].

Conversely, *Tetrastigma hemsleyanum* is only known in TDM for its ability to treat detumescence, while Bulang medicine recognizes its potential to promote hemostasis, stimulate blood circulation, and alleviate swelling. Further research demonstrates that *Tetrastigma hemsleyanum*, particularly its root tuber and whole herb, possesses additional pharmacological activities such as heat clearing and detoxification, blood circulation activation, pain relief, wind and phlegm dispelling, and efficacy against conditions like poisonous snakebites, whooping cough, bronchitis, pneumonia, pharyngitis, hepatitis, pediatric hyperthermia, and tumors [73–75]. Comparison to TDM illustrates the progressive nature of Bulang medicine. Further exploration of the various medicinal properties of medicinal plants may provide valuable insights for developing new drugs and advancing medical practice, contributing to a more comprehensive understanding of plants' medicinal efficacy and potential value for both traditional and modern medical practices.

## Conclusions

The study investigated the ethnobotanical knowledge of medicinal plants among Bulang people, evaluating the current status of research and utilization of their medicinal knowledge. A total of 60 species, 41 families and 59 genera of medicinal plants were utilized by Bulang people. Environmental changes are increasingly leading to the extinction of medicinal plants, which could contribute to people preferring modern Western medicine over traditional medicine. As the disappearance of these plants has the potential to reduce the availability of medicinal materials and limit the development of treatments, it also risks hindering the progress of scientific and medical research. It is, therefore, crucial to preserve these plants and their use by fostering sustainable harvesting practices, protecting habitats, and supporting research on their potential benefits. Notably, all medicinal plants used were mainly distributed in the wild, with the root being the most used part and the primary preparation method being decoction. Results of the study revealed that 41 diseases were treated with medicinal plants, with illnesses related to the digestive system being the most common. The most used plant species were those related to the motor system category.

A comparison between Bulang and Dai medicine revealed that 22 (36.67%) of the 60 plants investigated had more curative potential in Bulang medicine than Dai medicine. To further investigate the significant significance of medicinal plants, it is imperative to prioritize collaborative research efforts focused on the interplay between traditional ethnic remedies. The study also highlighted that the most significant medicinal values were in ethnomedicine closest to daily life, such as the therapeutic values of tea, ginger, and other staples. However, the medicinal values of some plants are gradually declining with environmental changes, and there is a growing concern that they may be forgotten or replaced by increasingly convenient western medicines. The decrease in the number of Bulang traditional herbalists was identified as the most significant threat to the development of Bulang medicine. In conclusion, the study provides essential insights into the rich ethnobotanical knowledge of Bulang people, highlighting the potential for further research to explore their medicinal plants' therapeutic values and safeguard their traditional medicinal knowledge.

## Data Availability

All data generated or analyzed during this study are included in this published article.

## Abbreviations

A: Analgesia
C: Circulatory system
D: Digestive system
E: Endocrine diseases
G: Gynecology
Gs: Genitourinary system
I: Immune system
M: Motor system
O: Other uses
R: Respiratory
Ethnic groups B: Bulang People
D: Dai People
HITBC: Xishuangbanna Tropical Botanical Garden Herbarium
NDT: Number of diseases treated
RMB: Renminbi
TCM: Traditional Chinese Medicine
UR: Use report
UV: Use value
ICF: Informant Consensus Factor
TDM: Traditional Dai Medicine
