# Correction: Ethnobotanical study on medicinal plants used by Bulang people in Yunnan, China

**DOI:** 10.1186/s13002-023-00622-3

**Published:** 2023-11-03

**Authors:** Hao Zhou, Jiaqi Zhang, Brian S. Kirbis, Zi Mula, Wei Zhang, Yinzhi Kuang, Qing Huang, Lun Yin

**Affiliations:** 1 School of Geography and Ecotourism, Southwest Forestry University, Kunming, 650224 Yunnan China; 2 Center for Integrative Conservation, Xishuangbanna Tropical Botanical Garden, Chinese Academy of Sciences, Menglun, 666303 Yunnan China; 3 University of Chinese Academy of Sciences, Beijing, 100049 China; 4 Ministry of Education Key Laboratory for Ecology of Tropical Islands, Key Laboratory of Tropical Animal and Plant Ecology of Hainan Province, College of Life Sciences, Hainan Normal University, Haikou, 571158 Hainan China; 5 Jing Hong, China; 6 Xishuangbanna Ancient Tea Plant Conservation and Development Association, Jing Hong, 666100 Yunnan China; 7 Southwest Ecological Civilization Research Center, National Forestry and Grassland Administration, Kunming, 650224 Yunnan China

**Correction: Journal of Ethnobiology and Ethnomedicine (2023) 19:38** **https://doi.org/10.1186/s13002-023-00609-0**

Following publication of the original article, the authors flagged two formatting errors: In the body of Fig. 10, ‘plants’ had been misspelled; throughout the manuscript, certain scientific names had been put in italics while, according to accepted conventions in botanical nomenclature, they should not have been. The published article has since been updated to correct these errors. The authors thank you for reading this erratum and apologize for any inconvenience caused.

